# High Glucose-Mediated Tyrosine Nitration of PI3-Kinase: A Molecular Switch of Survival and Apoptosis in Endothelial Cells

**DOI:** 10.3390/antiox7040047

**Published:** 2018-03-25

**Authors:** Sally L. Elshaer, Tahira Lemtalsi, Azza B. El-Remessy

**Affiliations:** Retinopathy Research, Augusta Biomedical Research Corporation Charlie Norwood VA Medical Center, Augusta, GA 30912, USA; dr_s_elshaer@mans.edu.eg or slelshaer@gmail.com (S.L.E.); tlemtalsi@augusta.edu (T.L.)

**Keywords:** peroxynitrite, tyrosine nitration, high glucose, PI3-kinase, apoptosis, endothelial cells, p38 MAPK, survival, Akt

## Abstract

Diabetes and hyperglycemia are associated with increased retinal oxidative and nitrative stress and vascular cell death. Paradoxically, high glucose stimulates expression of survival and angiogenic growth factors. Therefore, we examined the hypothesis that high glucose-mediated tyrosine nitration causes inhibition of the survival protein PI3-kinase, and in particular, its regulatory p85 subunit in retinal endothelial cell (EC) cultures. Retinal EC were cultured in high glucose (HG, 25 mM) for 3 days or peroxynitrite (PN, 100 µM) overnight in the presence or absence of a peroxynitrite decomposition catalyst (FeTPPs, 2.5 µM), or the selective nitration inhibitor epicatechin (100 µM). Apoptosis of ECs was assessed using TUNEL assay and caspase-3 activity. Immunoprecipitation and Western blot were used to assess protein expression and tyrosine nitration of p85 subunit and its interaction with the p110 subunit. HG or PN accelerated apoptosis of retinal ECs compared to normal glucose (NG, 5 mM) controls. HG- or PN-treated cells also showed significant increases in tyrosine nitration on the p85 subunit of PI3-kinase that inhibited its association with the catalytic p110 subunit and impaired PI3-kinase/Akt kinase activity. Decomposing peroxynitrite or blocking tyrosine nitration of p85 restored the activity of PI3-kinase, and prevented apoptosis and activation of p38 MAPK. Inhibiting p38 MAPK or overexpression of the constitutively activated Myr-Akt construct prevented HG- or peroxynitrite-mediated apoptosis. In conclusion, HG impairs pro-survival signals and causes accelerated EC apoptosis, at least in part via tyrosine nitration and inhibition of PI3-kinase. Inhibitors of nitration can be used in adjuvant therapy to delay diabetic retinopathy and microvascular complication.

## 1. Introduction

Exposure to hyperglycemia contributes to diabetes-associated microvascular and macrovascular complications. The Diabetic Complication Clinical Trial (DCCT) and the United Kingdom Prospective Diabetes Study (UKPDS) epidemiological studies correlated poor glycemic control with the development of vascular complications in patients with type 1 or type 2 diabetes mellitus [1,2]. These studies indicated that early exposure to hyperglycemia predisposes individuals to the development of diabetic complications, a phenomenon referred to as metabolic memory or the legacy effect [3]. It has been well-documented that high glucose-mediated oxidative and nitrative stress are integral to the development of diabetes-induced microvascular injury (reviewed in [4]). Microvascular endothelial cells (ECs) are sensitive targets of diabetic and hyperglycemia resulting in vascular injury [5]. Our previous work demonstrated that high glucose triggered formation of peroxynitrite in retinal EC cultures [6]. Diabetic retinopathy is broadly classified into two stages; a non-proliferative background stage, and the blinding proliferative retinopathy stage. By the end of the early non-proliferative stage, clinically significant ischemia takes place when a critical number of retinal capillaries become occluded and non-perfused [7]. Therefore, treatments that prevent or slow down retinal capillary cell death and consequent ischemia are critical for slowing progression of diabetic retinopathy into late sight-threatening proliferative stage.

Peroxynitrite, a highly reactive oxidant, can cause reduction of cellular antioxidant defenses, lipid peroxidation, and inhibition of key metabolic enzymes via nitration of the protein tyrosine residues or by oxidation of thiol pools [8]. Growing evidence supports the concept that covalent modification of various proteins by tyrosine nitration is associated with modification of biological functions (reviewed in [9]). Moreover, we have shown that increased formation of peroxynitrite mediates apoptosis in endothelial cells cultured in high oxygen [10], and in a model for oxygen-induced retinopathy [11] and in the diabetic retina [12].

It has been reported that retinal endothelial cells in diabetic patients and animals undergo accelerated death by a process consistent with apoptosis [13,14,15]. Paradoxically, high glucose stimulates expression of survival and angiogenic growth factors within retinal capillaries, suggesting uncoupling of survival signaling. Tyrosine nitration is a post-translational protein modification that has been postulated to alter protein function and contribute to metabolic memory and legacy effect [16]. Together, the pivotal role of tyrosine nitration in altering survival signal under diabetic conditions has prompted us to study the effects of high glucose-induced peroxynitrite on cell death in retinal EC cultures. The study attempted also to define the molecular mechanism by which peroxynitrite-mediated tyrosine nitration inhibits the survival signaling pathway.

## 2. Materials and Methods

### 2.1. Cell Culture

Retinal endothelial cells were isolated from bovine retinas as described previously [6]. The cells (passages 6–8) were grown to confluence and switched to serum free media, then treated in conditions of normal glucose (NG, 5 mM glucose) or high glucose (HG, 25 mM glucose) for 3 days, as described before [17]. Additional group of cells were treated with peroxynitrite 0.5 mM (Cayman, Ann Arbor, MI, USA) overnight (16 h), as described before [10]. Serum starvation is known to induce apoptosis in endothelial cells [18]. Stock concentrations of peroxynitrite were provided in 0.1 N NaOH. Inhibitors for peroxynitrite (FeTPPs) and epicatechin were obtained from Millipore-Sigma, (Saint Louis, MO, USA), and inhibitor for p38 MAPK (SB203580) was obtained from Calbiochem (La Jolla, CA, USA).

### 2.2. Caspase-3 Activity

The activity of caspase-3 enzyme was determined using a kit from R&D systems (Cat. #: K105-25, Minneapolis, MN, USA) according to the manufacturer’s instruction, as described earlier by our group [19]. Briefly, cells were lysed on ice for 10 min with lysis buffer provided with the kit. To 50 μL of cell lysate, 50 μL of 2X reaction buffer was added, followed by 5μL of caspase-3 fluorogenic substrate (DEVD-AFC). The mixture was incubated for 2 h at 37 °C, and fluorescence was measured with a fluorescent plate reader (BioTek Synergy2, Winooski, VT, USA) with an excitation of 400 nm and an emission of 505 nm. Results were normalized to non-starved EC cultures.

### 2.3. TUNEL Assay

ApopTag^®^ Fluorescein In Situ Apoptosis Detection Kit was used to determine cell death in EC cultures according to the manufacturer’s instructions (S7110, Millipore, Darmstadt, Germany). Cells were grown on coverslips and treated with high glucose or peroxynitrite as described previously [12]. EC cultures were counterstained with propidium iodide (PI) and cover-slipped with Vectashield (Vector Laboratories, Burlingame, CA, USA). Micrographs were captured at 20X by fluorescent microscope (AxioObserver.Z1; Zeiss, Jena, Germany).

### 2.4. Western Blotting Analysis

EC cultures were harvested after various treatments and lysed in modified radioimmunoprecipitation assay (RIPA) buffer (Cat# 20-188, Millipore-Sigma, Burlington, MA, USA) and 50 μg of total protein was separated on a 10–12% SDS-polyacrylamide gel by electrophoresis, transferred to nitrocellulose, and incubated with specific antibody. Antibodies for phospho-p38 MAPK, p38 MAPK, Akt, and Akt kinase were purchased from Cell Signaling Technology, Inc. (Beverly, MA, USA). The primary antibody was detected using a horseradish peroxidase-conjugated goat anti-mouse or anti-rabbit antibody (EMD, La Jolla, CA, USA) and enhanced chemiluminescence. The films were scanned, and the band intensity was quantified using ImageJ densitometry software version, and expressed as optical density (OD).

### 2.5. Immunoprecipitation

EC cultures were incubated in high glucose or peroxynitrite for the desired time in the presence or absence of peroxynitrite decomposition catalyst FeTPPs or nitration inhibitor, epicatechin. EC lysates were prepared, as described above, for immunoblotting. For PI3-kinase tyrosine nitration, 100 μg protein was incubated with p85. The precipitated proteins were analyzed by SDS-PAGE, and blotted with nitrotyrosine antibody or p85 for equal loading. To study the effects on the interaction of the regulatory subunit with the catalytic subunit of PI3-kinase, 100 μg protein was incubated with p110. Antibodies for nitrotyrosine, p85, and p110 subunits of PI3-kinase were purchased from Millipore (Millipore-Sigma, Burlington, MA, USA). For immunoprecipitation, signals were captured, and band intensities were measured using alphaEaseFC (Santa Clara, CA, USA)

### 2.6. Akt Kinase Assay

Lysates from HG- or PN-treated culture (100 μg) protein were incubated with immobilized Akt (Cell Signaling Technology, Danvers, MA, USA) monoclonal antibody-containing beads with gentle rocking at 4 °C overnight. The beads were washed then incubated in 40 μL kinase buffer supplemented with 200 μM ATP and 1 μg GSK-3 fusion protein for 30 min at 30 °C. The reaction was terminated with sample buffer. The supernatant was separated on a 12% SDS-PAGE gel, transferred to nitrocellulose membrane. Western immunoblotting was performed with anti-phopho-GSK-3α/β (Ser21/9) antibody, and total loading with anti-Akt antibody. Horseradish peroxidase-conjugated goat anti-rabbit antibody and enhanced chemiluminescence were used to detect the primary antibody. The films were scanned, and the band intensity was quantified using the National Institute of Health densitometry ImageJ software version.1 and expressed as optical density (OD).

### 2.7. Adenoviral Constructs

β-Galactosidase and C-terminal HA-tagged constitutively active Akt (Myr-Akt) were provided as a kind gift from Dr. David Fulton (Augusta University, Augusta, GA, USA) and generated as described previously [20]. EC cultures were infected with adenovirus containing the β-galactosidase and Myr-Akt. The virus was removed, and EC were left to recover for 12 h in complete medium. Western blot analysis using antibodies against total Akt (Cell Signaling Technology, Inc. Beverly, MA, USA) confirmed the overexpression in EC cultures transfected with Myr-Akt, but not in cells transfected with β-galactosidase.

### 2.8. Statistical Analysis

Results are expressed as mean ± SE, and the data was processed for statistical analysis. For experiments that examined apoptotic insult against control, one-way ANOVA was used. For experiments that examined effects of apoptotic insults (HG, PN) and treatments (Fe, Epi) against controls, two-way ANOVA was used, followed by Bonferroni post hoc multiple comparisons to assess significant differences among individual groups by GraphPad Software Version.6 (San Diego, CA, USA). Significance was defined at probability *p* < 0.05.

## 3. Results

### 3.1. High Glucose-Induced Peroxynitrite Induces Apoptosis in Retinal Endothelial Cells

**Figure 1:**
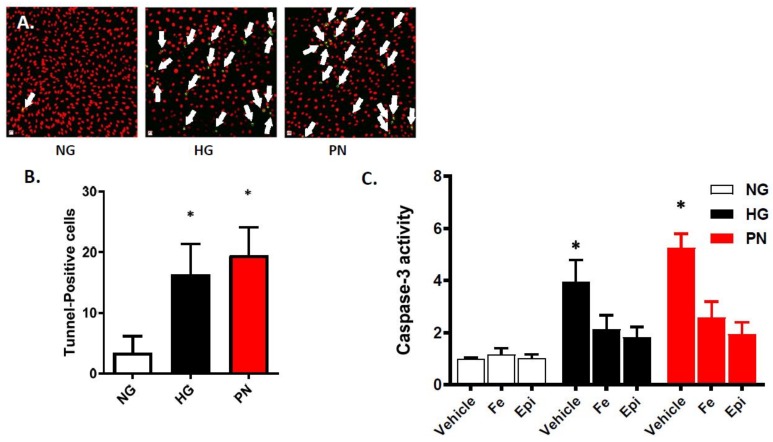
High glucose and peroxynitrite accelerated apoptosis in epithelial cell (EC) cultures. (**A**) Representative micrographs of TUNEL-positive nuclei (green, indicated by white arrows) in EC cultures counterstained with propidium iodide (red), and images were taken at 20× magnification. (**B**) Statistical analysis using one-way ANOVA showing significant increase in the number of TUNEL-positive cells in ECs treated with high glucose (HG, 25 mM) for 3 days or peroxynitrite (PN, 0.5 mM) for overnight, compared with normal glucose (NG) controls. (* *p* < 0.05, *n* = 6). (**C**) Statistical analysis of caspase-3 enzyme activity using two-way ANOVA showed significant interaction among examined groups. Treatment of EC cultures with HG (25 mM) for 3 days or PN (0.5 mM) overnight significantly increased activity of caspase-3 compared to NG. Co-treatment with the specific peroxynitrite decomposition catalyst FeTTPs (Fe, 2.5 μM) or the nitration inhibitor epicatechin (Epi, 100 μM) significantly reduced the increase in caspase-3 activity. (* *p* < 0.05, significant using Bonferroni test compared to the rest of the groups, *n* = 5–6).

Our previous studies showed that high glucose (HG) triggered significant increases in peroxynitrite formation in retinal EC at 3 and 5 days [6]. In the present study, we examined the effects of HG-induced nitrative stress (3-days) on EC survival in comparison to normal glucose (NG). Due to powerful apoptotic effects of exogenous peroxynitrite (PN), treatment was limited to overnight (16 h), as described previously [10]. As shown in Figure 1A,B, high glucose and peroxynitrite treatment accelerated EC death as indicated by significant increases in the number of TUNEL-positive cells compared to NG-controls. Caspase-3 is an intracellular cysteine protease that exists as a pro-enzyme, becoming activated during the cascade of intracellular signaling events that culminates in apoptosis. HG and PN significantly increased caspase-3 activity compared to NG controls (Figure 1C). Co-treatment with the specific peroxynitrite decomposition catalyst FeTPPs (Fe, 2.5 μM) or the epicatechin (Epi, 100 μM) significantly reduced cell death in high glucose and peroxynitrite-treated cells. Epicatechin, a dietary flavenol, has been shown to selectively blunt the nitrating effect of peroxynitrite on tyrosine residues [21,22]. These findings suggest that the effect of HG in accelerating apoptosis in retinal EC cultures likely involves PN-mediated alteration of cell survival pathways.

### 3.2. High Glucose and Peroxynitrite-Mediated Tyrosine Nitration of p85 Subunits Causes PI3-Kinase Dysfunction

**Figure 2:**
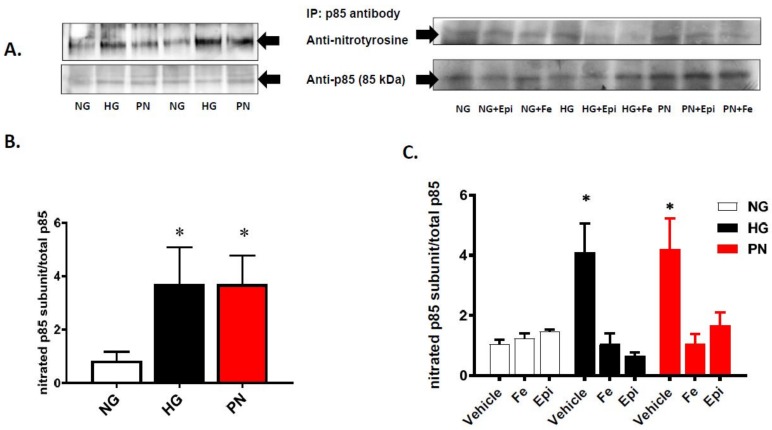
High glucose and peroxynitrite cause tyrosine nitration of the p85 subunit in ECs. (**A**) Representative blots of EC cultured in normal glucose (NG, 5 mM), high glucose (HG, 25 mM) for 3 days, or peroxynitrite (PN, 0.5 mM) overnight (left panel), alone or in the presence or absence of the specific peroxynitrite decomposition catalyst FeTTPs (Fe, 2.5 μM) or the nitration inhibitor epicatechin (Epi, 100 μM) (right panel). EC lysates were immune-precipitated with antibody against p85 subunit, and immunoblotted with antibodies that detect nitrotyrosine (NY) or anti-p85 s-subunit as loading control. (**B**) Statistical analysis of densitometry ratio of tyrosine-nitrated (NY) p85 subunit to total p85 using one-way ANOVA showed significant tyrosine nitration of p85 subunit in HG- and PN-treated cultures compared to normal glucose (* *p* < 0.05 vs. NG, *n* = 4). (**C**) Statistical analysis of densitometric ratio of tyrosine-nitrated (NY) p85 subunit to total p85 using two-way ANOVA showed significant interaction among examined groups. There was significant increase of tyrosine nitration of p85 subunit in HG- or PN-treated EC lysates compared to NG controls. Inhibiting tyrosine nitration using FeTTPs or epicatechin significantly reduced tyrosine nitration in HG- or PN-treated cells (* *p* < 0.05 versus rest of the groups, significant using Bonferroni test compared to the rest of the groups, *n* = 4–5).

**Figure 3:**
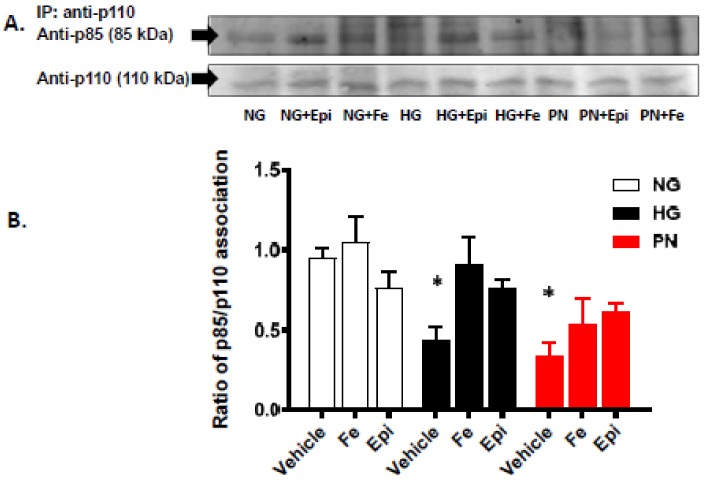
High glucose and peroxynitrite inhibit association between p85 and p110 subunits of PI3- kinase. (**A**) Representative blot of the catalytic subunit (p110) immunoprecipitation showing protein–protein interaction between p110 and the regulatory subunit (p85) that was decreased under high glucose (HG, 25 mM) for 3 days, or peroxynitrite (PN, 0.5 mM) overnight, as compared to normal glucose (NG, 5 mM) but restored back in the presence of FeTTPs (Fe, 2.5 μM) or epicatechin (Epi, 100 μM). (**B**) Statistical analysis of the ratio of p85 subunit and p110 subunit using two-way ANOVA showed significant impact of apoptotic insult and also for treatment compared to insult. EC cultures treated with HG or PN showed significant decrease in association of p85 subunit and p110 subunit compared to NG. The association between p85 and p110 was restored in cultures co-treated with the specific peroxynitrite decomposition catalyst FeTTPs (Fe, 2.5 μM) or the nitration inhibitor epicatechin (Epi, 100 μM) (* *p* < 0.05 versus vehicle, significant using Bonferroni test compared to the rest of the groups, *n* = 4–5).

Previous studies have shown that the p85 regulatory subunit of PI3-kinase is a sensitive target for peroxynitrite-induced tyrosine nitration [11,23,24]. Our immunoprecipitation assay showed that EC cultured in HG or treated with PN showed a significant increase in tyrosine nitration of p85 compared to EC cultured in normal glucose (Figure 2A, B). The increase in tyrosine nitration was markedly attenuated by the specific peroxynitrite decomposition catalyst FeTTPs (Fe, 2.5 μM) and by the specific nitration inhibitor epicatechin (Epi, 100 μM) (Figure 2B,C). In order to confirm the inhibitory effect of tyrosine nitration on PI3-kinase function, we determined the effects of HG and exogenous PN on the association between the regulatory p85 subunit and the catalytic p110 subunit in response to peroxynitrite, and in the presence or absence of nitration inhibitors. Treatment with HG or PN caused dissociation of p85 from p110 as the band for p85 could no longer be detected in the immune-precipitates (Figure 3A). Meanwhile, re-probing the blots with antibody against p110 subunit revealed the presence of p110 protein band. Treatments with FeTPPs or epicatechin restored the association between p85 and p110 in HG- or PN-treated EC cultures, and did not affect normal glucose controls. These results suggest that PN-mediated tyrosine nitration of p85 could alter the cell survival responses mediated normally by PI3-kinase activation.

### 3.3. High Glucose-Induced Peroxynitrite Inhibits Akt and Activates P38 MAPK in ECs

**Figure 4:**
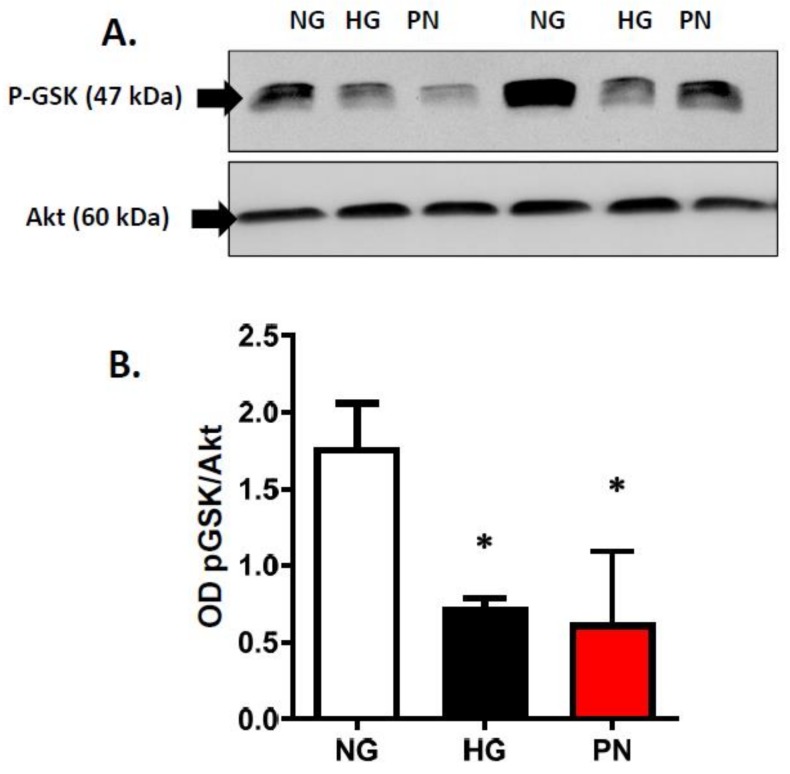
High glucose and peroxynitrite impaired Akt kinase activity. (**A**) Representative Western blot images of p-GSK-3α/β (Ser21/9) associated with total Akt that were immune-precipitated from EC cultures treated with normal glucose (NG, 5 mM), high glucose (HG, 25 mM) for 3 days, or peroxynitrite (PN, 0.5 mM) for overnight. (**B**) Statistical analysis using one-way ANOVA showed that HG or PN significantly inhibited survival signal evident by significant decrease in GSK-3α/β phosphorylation compared with NG. (* *p* < 0.05 versus NG, *n* = 3–4).

**Figure 5:**
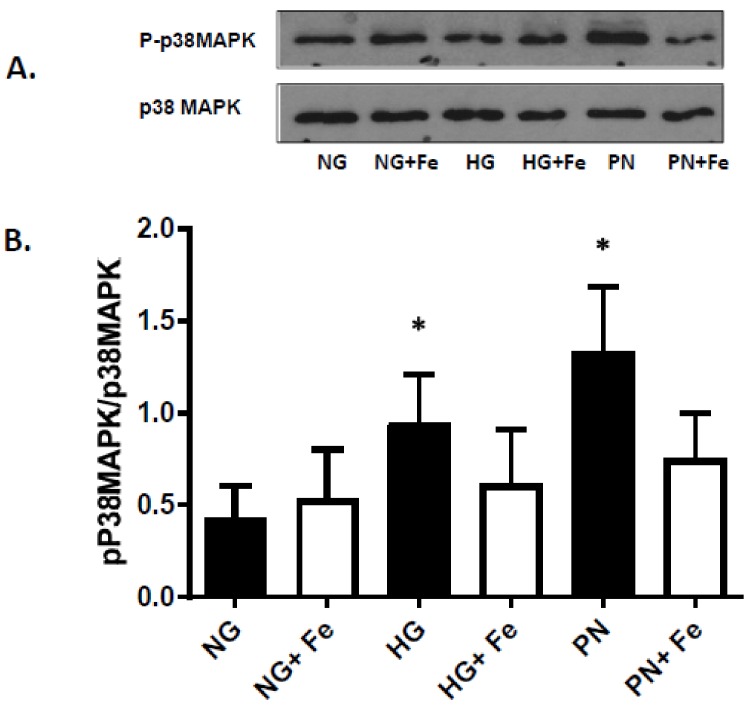
High glucose and peroxynitrite increased p38 MAPK activation that was reversed by FeTTPs. (**A**) Representative Western blot of total and phosphorylated p38 MAPK in lysates of retinal EC cultures treated with normal glucose (NG), high glucose (HG, 25 mM) for 3 days, or peroxynitrite (PN, 0.5 mM) overnight. **B.** Statistical analysis using two-way ANOVA showed significant impact of apoptotic insult and treatment effect. EC treated with HG or PN caused significant increase in p38 MAPK activation compared to NG. This effect was significantly reduced by co-treatment with FeTTPs (Fe, 2.5 μM) in HG- or PN-treated cultures. (* *p* < 0.05, significant using Bonferroni test compared to the rest of the groups, *n* = 5–6).

In response to survival factors, activation of PI3-kinase/Akt pathway is essential for EC survival, whereas dysfunction of PI3-kinase can result in activation of p38 MAPK and EC death. Next, we examined whether HG and PN-mediated nitration and dysfunction of PI3 kinase can result in alterations of the pro-survival Akt pathway or activation of the proapoptotic p38 MAPK pathway. As shown in Figure 4A, HG and PN significantly reduced the activity of Akt kinase activity evident by significant decreases in phosphorylation of its downstream target GSK-3. Meanwhile, re-probing the blots with Akt showed comparable levels of Akt protein. Moreover, treatment with HG or PN was associated with significant increases in phosphorylation of p38 MAPK (representative (Figure 5A) and bar graph (Figure 5B)). Co-treatment of EC maintained in HG or PN with the specific peroxynitrite decomposition catalyst FeTPPs attenuated the increases in p38 MAPK phosphorylation (Figure 5A,B). These findings suggest that nitration-mediated dysfunction of PI-3kinase is associated with activation of proapoptotic p38 MAPK signal and inhibition of the survival Akt signal in EC.

### 3.4. Overexpression of Active Akt Restores EC Cultures’ Survival

**Figure 6:**
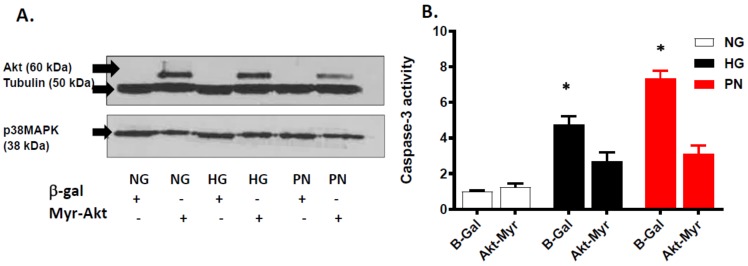
Overexpression of Myr-Akt attenuates proapoptotic effect of high glucose and peroxynitrite. (**A**) Representative Western blot showing selective expression of Akt in retinal EC cultures transfected with constitutively active Akt (Myr-Akt), but not in cultures transfected with adenovirus construct of β-galactosidase (β-Gal). Overexpression of Myr-Akt decreased expression of p38 MAPK. These observations were consistent in ECs cultured in normal glucose (NG, 5 mM), high glucose (HG, 25 mM) for 3 days, or peroxynitrite (PN, 0.5 mM) for overnight. (**B**) Statistical analysis of caspase-3 activity using two-way ANOVA showed significant effect of apoptotic insult and for treatment. Overexpression of Myr-Akt significantly reduced HG- or PN-induced increase in caspase-3 activity compared to EC cultures transfected with β-Gal (* *p* < 0.05, significant using Bonferroni test compared to the rest of the groups, *n* = 5).

To confirm the inhibitory role of PI3-kinase dysfunction in inhibiting pro-survival effects of Akt under HG or PN, we used adenoviral-mediated gene transfer of constitutively active Akt (Myr-Akt) into retinal EC cultures. A representative image of Western blot confirmed the selective overexpression of Akt in EC cultures transfected with Myr-Akt, but not in cells transfected with β-galactosidase (Figure 6A). Interestingly, the anti-apoptotic effect of Myr-Akt was associated with significant decreases in p38 MAPK expression in HG- or PN-treated EC compared to cultures infected with β-galactosidase constructs. Figure 6B shows that overexpression of the constituently activated Akt (Myr-Akt) significantly mitigated HG- or PN-induced increase in caspase-3 activity, as compared to EC transfected with β-galactosidase constructs. The above data imply that when the Akt pro-survival pathway is activated, the proapoptotic of p38 MAPK-driven pathway is downplayed.

### 3.5. Inhibition of p38 MAPK Restores Cell Survival in Retinal EC Cultures

**Figure 7:**
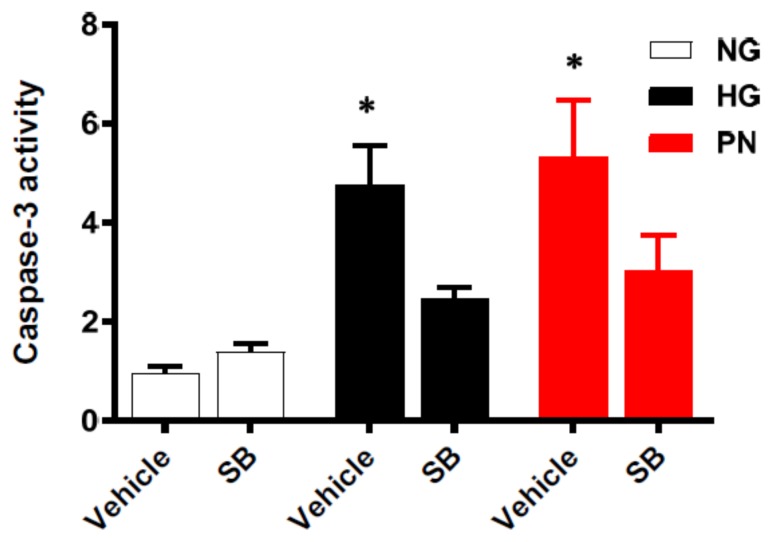
Inhibition of p38 MAPK using SB203580 attenuated high glucose and peroxynitrite-induced apoptosis in retinal EC cultures. Retinal EC cultures were co-treated with normal glucose (NG), high glucose (HG, 25 mM) for 3 days, or peroxynitrite (PN, 0.5 mM) for overnight in the presence or absence of the p38 MAPK inhibitor; SB203580 (25 μM). Statistical analysis using two-way ANOVA showed significant impact of both apoptotic insult and treatment. Inhibiting p38 MAPK significantly attenuated caspase-3 activity in HG- or PN-treated EC cultures compared with controls. (* *p* < 0.05 versus vehicle, significant using Bonferroni test compared to the rest of the groups, *n* = 5).

To confirm the role of p38 MAPK activation in the proapoptotic effects of HG or PN, we treated the cells with the specific p38 MAPK inhibitor; SB203580 (25 μM). As shown in Figure 7, treatment of HG or PN induced apoptosis evidenced by significant increases in caspase-3 activity. Co-treatment with the p38 MAPK inhibitor significantly reduced the apoptosis induced in HG-maintained or PN-treated cultures. Treatment with SB203580 did not affect cell death in the normal glucose control. It is interesting that protective effect of SB203580 was more prominent in HG-treated cultures than in PN-treated cultures.

## 4. Discussion

**Figure 8:**
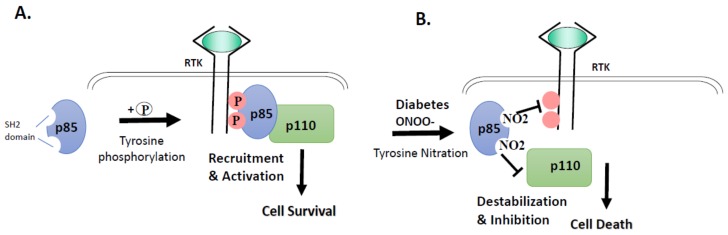
Conceptual frame of the study results. (**A**) Under normal physiological conditions, proper phosphorylation of regulatory subunit of PI3-kinase: p85 by receptor tyrosine kinases (RTK) takes place, resulting in strong association with the catalytic subunit p110, and initiation of cell survival signal. (**B**) Under diabetic or nitrative stress conditions, nitration of p85 subunit—instead of phosphorylation—occurs, resulting in its dissociation from p110 subunit and dysfunction of PI3-kinase and initiation of cell death signal.

Tyrosine nitration is a post-translational protein modification that can result in dramatic changes in protein structure and can modulate protein function (reviewed in [8]). In this study, we demonstrate that tyrosine nitration of the PI3-kinase p85 subunit inactivates the survival signaling pathway in ECs cultured in HG or PN (Figure 1, Figure 2 and Figure 3). These effects are blocked by the specific peroxynitrite decomposition catalyst FeTPPs, and by the specific nitration inhibitor epicatechin. We show also that the proapoptotic effect of HG or PN is associated with an imbalance between Akt and p38 MAPK activation (Figure 4 and Figure 5). Inhibiting p38 MAPK or overexpression of the constitutively active Akt masks the proapoptotic effect of HG or PN, and restores survival function in retinal EC cultures (Figure 6 and Figure 7). The present study examined the impact of tyrosine nitration to confer inhibition of PI-3kinase/Akt, and activation of p38 MAPK pathway, resulting in apoptosis. Here, we provide evidence that inhibition of PI3-kinase by peroxynitrite-mediated tyrosine nitration triggers a molecular switch, resulting in reducing Akt activation, activating the proapoptotic p38 MAPK pathway, and eventually, cell death. A schematic presentation of these findings and the proposed mechanism by which tyrosine nitration of p85 subunit is serving as molecular switch between survival and apoptosis are illustrated in Figure 8.

Peroxynitrite, the combination product of nitric oxide and superoxide anion, is a powerful and short-lived free radical formed in vivo, that can directly react with different biomolecules by nitration and thiol oxidation (reviewed in [8]). When one molecule of superoxide-anion is formed, it may undergo the superoxide anion dismutase (SOD)-catalyzed dismutation reaction to hydrogen peroxide or interact with nitric oxide in a much faster reaction [25]. While superoxide anion and hydrogen peroxide-mediated oxidative effects are widely studied and well-documented [26,27], peroxynitrite remains a more relevant biological mediator of oxidative effects of both superoxide anion and nitric oxide in biological systems. Diabetes-mediated vascular dysfunction is highly linked to oxidative and nitrative stress [6,28]. Significant increases in tyrosine nitration have been demonstrated in plasma from diabetic patients [29], retinas from diabetic patients [12], and in experimental diabetes [30,31,32,33]. Previous in vitro work demonstrated that the p85 regulatory subunit of PI3-kinase is a susceptible target for peroxynitrite-induced tyrosine nitration [23]. Thus, we tested the hypothesis that HG-mediated apoptosis in EC cultures involves the action of peroxynitrite-mediated nitration and inhibition of the survival signal pathway. Indeed, EC cultures treated with HG or PN showed significant increases in tyrosine nitration of the regulatory p85 subunit and dysfunction of PI3-kinase (Figure 2 and Figure 3). The inhibitory effect of nitration of p85 is evident by dramatic decreases in the association of the p85 subunit with the catalytic p110 subunit, as well as by significant decreases in Akt kinase activity.

Moreover, decomposing peroxynitrite (FeTPPs) and inhibiting tyrosine nitration (epicatechin) attenuated tyrosine nitration of p85 subunit and restored the interaction between the regulatory p85 subunit and the catalytic p110 subunit of PI3-kinase and survival promoting activity. FeTPPs, a selective peroxynitrite scavenger, is an iron porphyrin complex that catalytically isomerizes peroxynitrite into nitrate, while epicatechin is a natural flavonoid that selectively blocks nitration reactions, which does not affect the antioxidant defense [11,34]. Of note, our prior in vivo work demonstrated protective effects of FeTPPs and epicatechin in experimental diabetes and ischemic retinopathy [11,12,34]. Our findings showing the inhibitory effect of tyrosine nitration of p85 subunit resulting in vascular cell death lend further support to prior reports that showed the inhibitory effect of tyrosine nitration of p85 subunit and vascular cell death in oxygen-induced retinopathy [11] and nitration of p85 in brain of diabetic animals [24]. Additional targets were identified, including tyrosine nitration and inhibition of the nerve growth factor (NGF) survival receptor, TrkA, resulting in neurodegeneration in experimental diabetes [12,34] and nitration of actin, resulting in loss of vascular tone in smooth muscles [35]. These results confirm the relationship between nitration of p85, the decreases in Akt activity, and the proapoptotic effects of high glucose and peroxynitrite.

HG- and PN-induced apoptosis was evident by TUNEL and caspase-3 enzyme activity. The proapoptotic effects of HG and PN were associated with activation of p38 MAPK and decreases in Akt kinase activity. It is interesting that expression of constitutively active Akt (Myr-Akt) masked the proapoptotic effects of high glucose and exogenous peroxynitrite in treated EC cultures (Figure 6). In addition, inhibition of p38 MAPK also rescued retinal EC cultures from accelerated cell death (Figure 7). The protective effects of SB203580 were more prominent in high glucose cultures than in peroxynitrite-treated cells, probably because of the magnitude of peroxynitrite-induced cell death. Our results are in agreement with previous reports in other vascular cells [11,36,37]. Furthermore, the anti-apoptotic effect of Myr-Akt was associated with significant inhibition of p38 MAPK. These findings lend further support to a prior study that demonstrated that inhibition of p38 activation is mediated through phosphorylation and inhibition of MEKK3 by Akt in aortic EC cultures [18]. Similar results were reported [36] in HUVEC in response to angiopoietin-1 and in cardiac microvascular EC cultures in response to IL-18 [38], suggesting that the inhibitory influence of Akt on p38 MAPK is a general phenomenon in EC cultures. Moreover, similar findings of the mutual regulatory effects of Akt and p38 MAPK were observed in breast cancer cells [39].

Uncontrolled diabetes is characterized by hyperglycemia that has been shown to drive oxidative and nitrative stress [6,28] and to trigger EC death [14,28]. Paradoxically, HG also stimulates expression of multiple survival factors, including vascular endothelial growth factor (VEGF), NGF, and fibroblast growth factor (FGF) [12,40,41,42,43]. Under physiological conditions, survival factors normally mediate its signal via activation of ligand-mediated receptor tyrosine kinase (RTK). As illustrated in Figure 8, activation of regulatory subunit of PI3-kinase, p85, results in strong association with the catalytic subunit, p110, and initiation of cell survival signal under physiological conditions. By contrast, under diabetic and/or pro-oxidative conditions, tyrosine nitration of the regulatory p85 subunit will prevent its subsequent activation, and lead to dysfunction of PI3-kinase. The inhibitory effect of nitration of p85 is evident by dramatic decreases in the association of the regulatory p85 subunit with the catalytic p110 subunit, as well as by significant decreases in Akt kinase activity. In conclusion, our report is the first to elucidate the causal and inhibitory effects of tyrosine nitration of regulatory p85 subunit leading to inactivation of the pro-survival effects of Akt, and activation of the proapoptotic effect of p38MAPK in retinal EC cultures. This proposed pathway may provide a conceptual frame to explain the paradox of increased production of growth factors and uncoupling of its survival effects under diabetic conditions. Thus, therapeutic strategies to restore survival signals, rather than to target growth factors, should be considered to combat diabetic complications.

## 5. Conclusions

While tight glycemic control can delay microvascular complications, most diabetic patients experience uncontrolled hyperglycemia that predisposes them to the development of diabetic complications. Exposure to HG promotes expression of survival and angiogenic factors, such as VEGF and FGF, however, HG-induced PN inactivates pro-survival function in retinal EC cultures and triggers apoptosis. A schematic representation of the proposed mechanism is shown in Figure 8. The findings that decomposing peroxynitrite and inhibiting tyrosine nitration restored survival function open the door for new therapeutic strategies. Preventing tyrosine nitration and restoring protein function in response to high glucose and diabetes will be essential to interrupt the metabolic memory and legacy effect. Nitration inhibitors, including epicatechin, can be attractive in adjuvant therapy, in addition to anti-hyperglycemic drugs, in controlling early diabetic microvascular complication.
